# First person – Vranda Garg

**DOI:** 10.1242/dmm.052403

**Published:** 2025-04-28

## Abstract

First Person is a series of interviews with the first authors of a selection of papers published in Disease Models & Mechanisms, helping researchers promote themselves alongside their papers. Vranda Garg is first author on ‘ Patient-specific mutation of contact site protein Tomm70 causes neurodegeneration’, published in DMM. Vranda conducted the research described in this article while a PhD student in Dr Bart Geurten and Dr Roland Dosch's lab at Georg-August-University Göttingen, Göttingen, Germany, and is now a postdoc in the lab of Dr Eric Samarut at University of Montreal Hospital Research Centre, Montreal, Canada, investigating the molecular and cellular mechanisms underlying neurodegenerative and neurological disorders using zebrafish models.


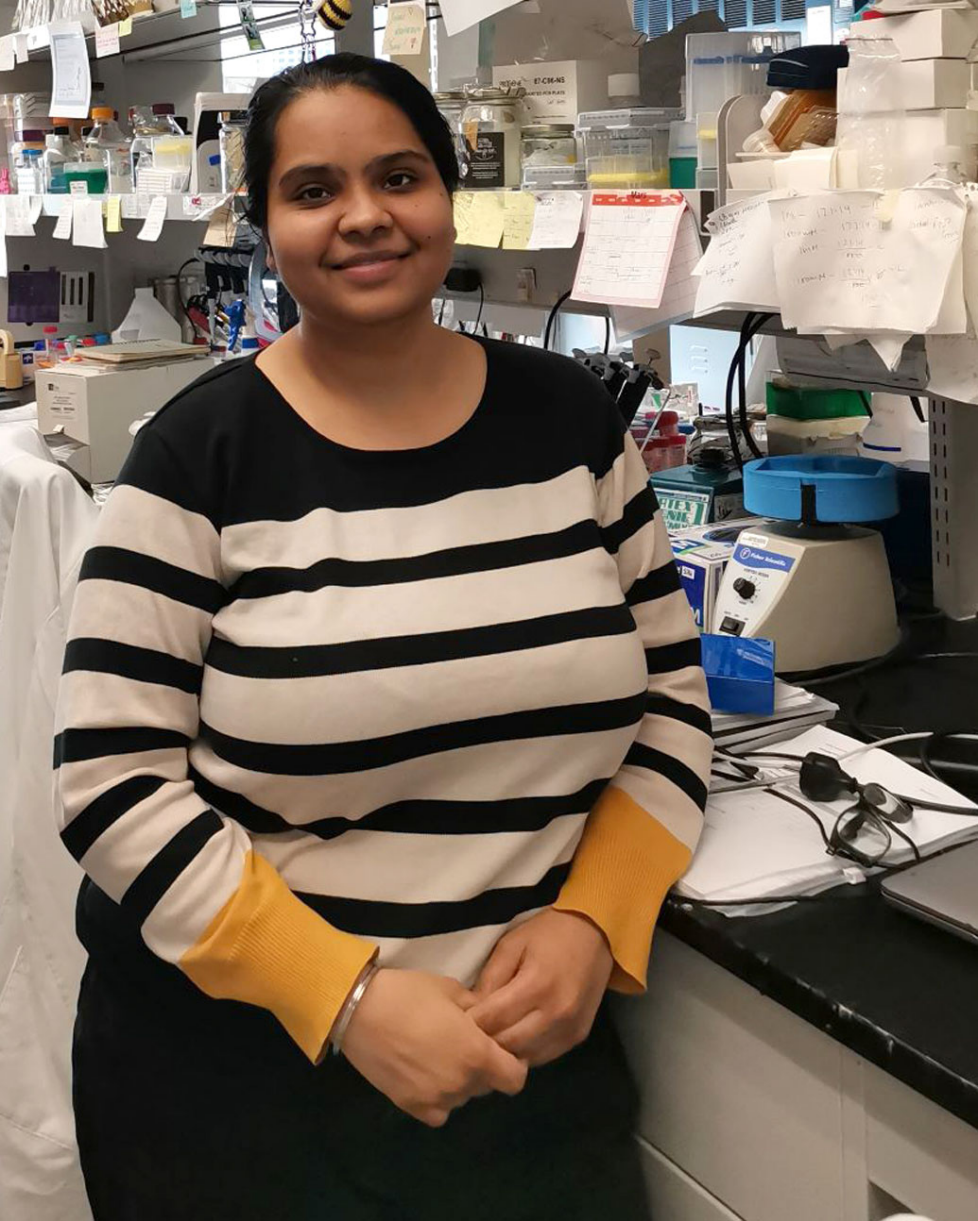


**Vranda Garg**

**Who or what inspired you to become a scientist?**

My curiosity to understand how the human brain works and what goes wrong in diseases proved to be my biggest motivation to pursue science. Until my Masters, I studied a variety of subjects and enjoyed learning about various aspects of biology, but my first interaction with my PhD supervisors Dr Bart Geurten and Dr Roland Dosch was a game changer for me. They introduced me to the field of zebrafish disease modelling and rare neurological disorders. I was fascinated by the fact that more than 88 different genes are involved in the pathogenesis of a rare neurodegenerative disorder and there is no clearly defined mechanism of pathogenesis. Therefore, I decided to work on this topic for my PhD, and that's how my scientific career began.

**What is the main question or challenge in disease biology you are addressing in this paper? How did you go about investigating your question or challenge?**

Hereditary spastic paraplegia (HSP) is a rare, complex, genetic disorder mainly characterised by the axonopathy of the longest corticospinal tract neurons, leading to progressive spasticity and weakness of the lower limbs. Until now, over 88 genes and 100 distinct spastic gait disease loci are known to be associated with HSP, and this represents only about 50% of the total, suggesting that many more genes remain to be discovered. The goal of my PhD leading to this paper was to define *TOMM70*, a mitochondrial outer membrane gene, as causative of HSP. A patient-specific mutation in *tomm70* in zebrafish leads to the change of a highly conserved amino acid, isoleucine, to threonine. I conducted behavioural, electrophysiological, electron microscopy, primary neuronal culture and immunocytochemistry, lipidomics and overexpression experiments to understand the effects of this mutation and ultimately define *TOMM70* as a novel gene involved in HSP pathogenesis.

**How would you explain the main findings of your paper to non-scientific family and friends?**

I studied a disease called HSP, which passes from parents to children. Patients with HSP have difficulty with walking, eventually requiring a wheelchair. There are no effective medicines available to treat the disease. There are many genes affected and many more still need to discovered. My task was to understand how one base change in DNA could cause walking problems and how it exactly happens. With my research, I found that zebrafish carrying the same DNA change as humans cannot swim constantly and have difficulties in bending their body. I also found that the gene *tomm70*, which has this one base change, was absent from axons and dendrites, parts of brain cells, and that Tomm70 partially lost its connection with its partner protein due to the one base change. With all this information, I identified *TOMM70* as a new gene involved in HSP.

My study provides evidence of the involvement of *TOMM70* in neurodegenerative disorders, particularly HSP.

**What are the potential implications of these results for disease biology and the possible impact on patients?**

My study provides evidence of the involvement of *TOMM70* in neurodegenerative disorders, particularly HSP. HSP patients are often misdiagnosed with amyotrophic lateral sclerosis or spinal muscular atrophy due to lack of information about the genes involved in the disorder. This study identifies *TOMM70* as a novel gene for HSP, which will help the better diagnosis of patients with this debilitating disorder.

**Figure:**
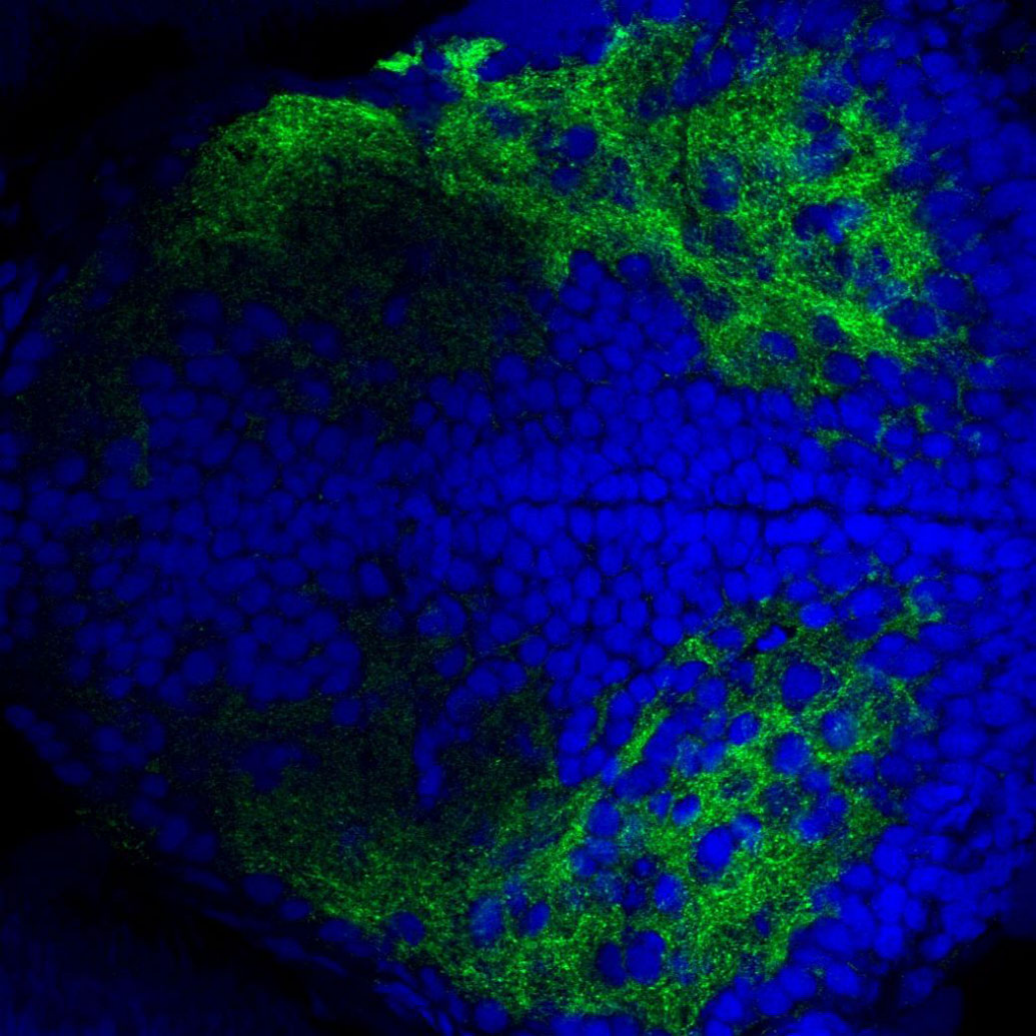
Transverse section of a zebrafish larval forebrain (6 days post-fertilisation) stained for glutamatergic synapses and nuclei using anti-Vglut1 antibody (green) and DAPI (blue), respectively.

**Why did you choose DMM for your paper?**

The theme of my paper perfectly aligns with the scope of DMM. Also, DMM is a highly reputed journal in this field. I believe that the followers and readers of this journal will also consider reading my work.

**Given your current role, what challenges do you face and what changes could improve the professional lives of other scientists in this role?**

I am an early-career postdoctoral scientist who wants to continue in academia and to start my own group in the next 4-5 years. Academia is like a vicious circle – more good publications in peer-reviewed, high-impact journals lead to higher chances for one to get funds to start and run a lab and permanent positions in universities and research institutes. This is also the biggest challenge for me right now. I think the availability of more funding options and academic positions can reduce the pressure not only on me but also on other postdoctoral scientists who want to transition to academic positions after finishing their postdoctoral studies.

**What's next for you?**

For almost 1.5 years, I have been pursuing my postdoctoral research in the lab of Dr Eric Samarut in the University of Montreal Hospital Research Centre, Montreal. Currently, I have two projects, one on epilepsy and another on HSP, both using a zebrafish model. I have decided to continue in academia, and, after finishing my postdoctoral studies in the next 3-4 years, I am planning on moving back to India and setting up my own lab to continue to work on rare neurological and neurodegenerative disorders using zebrafish as a model.

**Tell us something interesting about yourself that wouldn't be on your CV**

When I am not in the lab, I like to watch movies and dramas and to listen to music. I also like to travel and enjoy different types of cuisines. I also have a strong passion for social work and like to put my efforts into making the world a better place.
